# Assessment of anxiety risk association with PAHs and aluminum co-exposure in an aluminum production facility

**DOI:** 10.1186/s42506-026-00233-2

**Published:** 2026-09-17

**Authors:** Mohammad R. Zare, Hadi E. S. Kakhaki, Mehdi Zare, Mousa S. Ahmadi, Mehdi B. Ardakani

**Affiliations:** 1 Department of Environmental Health Engineering, School of Health, Larestan University of Medical Sciences, Larestan, Iran; 2 Department of Occupational Medicine, School of Medicine, Hormozgan University of Medical Sciences, Bandar Abbas, Iran; 3 Department of Occupational Health Engineering, Faculty of Health, Hormozgan University of Medical Sciences, Bandar Abbas, Iran; 4 Department of Biology and Vector Control, Department of Public Health, Social Determinants in Health Promotion Research Center, Research Institute for Health, Hormozgan University of Medical Sciences, Bandar Abbas, Iran

**Keywords:** Benzo[a]pyrene, 1-Hydroxypyrene, Aluminum, Occupational exposure, Anxiety, Aluminum industry, Neurotoxicity

## Abstract

**Background:**

Workers in aluminum production plants are occupationally exposed to polycyclic aromatic hydrocarbons (PAHs), including the neurotoxicant benzo[a]pyrene (BaP), as well as to aluminum dust and fumes. While the physical health effects are established, the psychological impact, particularly on anxiety, remains less explored. This study investigated the associations of urinary 1-hydroxypyrene (1-OHP), a biomarker of PAHs exposure, and urinary aluminum levels with anxiety symptoms in aluminum workers.

**Methods:**

In this cross-sectional study, 260 participants (130 exposed workers and 130 controls) from an aluminum plant were enrolled. Urinary aluminum concentration was measured using inductively coupled plasma mass spectrometry (ICP-MS). Anxiety was assessed using the Beck Anxiety Inventory (BAI), and job stress was evaluated using the Job Content Questionnaire (JCQ). BaP exposure was assessed using personal air sampling following NIOSH Method 5515. Urinary 1-OHP levels were quantified using high-performance liquid chromatography (HPLC). Group comparisons, Pearson correlation, and multiple linear regression analyses were performed using SPSS software version 22.

**Results:**

The exposed group had significantly higher anxiety scores (10.74 ± 6.18) than the control group (8.88 ± 5.28). Urinary aluminum concentration was significantly higher in the exposed group (33.45 ± 16.20 µg/g creatinine) compared to controls (7.35 ± 3.99 µg/g creatinine). In the final regression model, which included total job stress as an independent variable alongside age, job experience, and smoking, urinary 1-OHP (β = 0.204), urinary aluminum (β = 0.177), smoking (β = 0.202), and total job stress (β = 0.295) were all significantly associated with anxiety, explaining 38.4% of the variance (R² = 0.384).

**Conclusion:**

The internal dose of PAHs, measured via urinary 1-OHP, and aluminum body burden, measured via urinary aluminum, are significantly associated with higher anxiety symptoms. Notably, job stress also independently was associated with anxiety in this cohort. This highlights the potential neurotoxic impact of combined PAH and aluminum exposure on mental health and suggests the need for integrated psychological and biological monitoring in high-risk occupations.

**Supplementary Information:**

The online version contains supplementary material available at https://doi.org/10.1186/s42506-026-00233-2.

## Introduction

Polycyclic aromatic hydrocarbons (PAHs) are a class of organic compounds composed of multiple fused benzene rings, characterized by their environmental persistence and significant health risks [1]. These compounds are primarily generated from the incomplete combustion or pyrolysis of organic materials such as fossil fuels, biomass, and tobacco [2]. PAHs are ubiquitous environmental pollutants, found in various media including air, water, and soil, where they can accumulate due to their hydrophobic nature [3].

Among PAHs, benzo[a]pyrene (BaP) is one of the most extensively studied due to its high toxicity and well-characterized carcinogenic and mutagenic properties [3, 4]. BaP is often used as an indicator for the broader class of PAHs in environmental monitoring. Upon entering the body, BaP is metabolized into a highly reactive diol epoxide (BPDE) that forms DNA adducts, leading to mutations and malignant transformations [5]. Consequently, exposure to BaP and other PAHs has been linked to various adverse health outcomes, including respiratory diseases, cardiovascular disorders, and cancers [1].

Workers in industrial environments, such as aluminum production plants, face a significant risk of PAHs exposure, primarily through the inhalation of contaminated air and dermal contact with substances containing these compounds [6]. In the aluminum industry, BaP is generated as a byproduct of high-temperature processes, notably in smelting operations using Soderberg technology, where it is often found in the highest concentrations among PAH mixtures [7]. A large cohort study of aluminum smelter workers confirmed that chronic exposure to elevated levels of PAHs is a major occupational health concern, leading to potential long-term physical consequences [7].

Benzo[a]pyrene (BaP) is metabolized in the body into a variety of metabolites through complex pathways [5]. Unlike pyrene, BaP itself does not result in 1-hydroxypyrene (1-OHP) as a metabolite. Instead, 1-OHP is the primary metabolite of pyrene, another PAH commonly found alongside BaP in environmental and occupational mixtures [8]. Despite this, the measurement of urinary 1-OHP has been widely adopted as a practical, non-invasive biomarker for assessing recent exposure to PAH mixtures as a whole, due to the high correlation of pyrene with other PAHs in these mixtures and the relative ease of measuring 1-OHP [9]. Research has shown that urinary 1-OHP levels correlate with airborne concentrations of PAHs and can effectively monitor exposure levels in occupational settings like aluminum production [10].

In addition to PAHs, workers in aluminum production are also exposed to significant levels of aluminum dust and fumes. Aluminum is released during the electrolytic reduction process, particularly in Soderberg smelters, where it becomes aerosolized and inhalable [11]. Urinary aluminum has been validated as a biomarker of recent aluminum exposure in occupational settings, reflecting the integrated body burden from inhalation and dermal routes [12]. Chronic aluminum exposure has been associated with neurotoxicity, including cognitive impairment and disruption of neurotransmitter systems [13].

In addition to its well-established physical health effects, emerging evidence indicates that BaP can exert neurotoxic and psychological impacts [3]. The mechanisms underlying these effects are thought to involve the induction of oxidative stress and inflammatory responses within the central nervous system [14, 15]. For instance, acute BaP exposure in astrocytic cells has been shown to significantly increase the production of pro-inflammatory cytokines and elevate markers of oxidative stress [14]. Furthermore, developmental exposure to BaP can disrupt the architecture of key neuronal networks [16]. In animal models, chronic BaP administration has been demonstrated to induce anxiety-like behaviors, which were associated with epigenetic changes and down-regulation of neural receptors [17]. Aluminum also exhibits similar neurotoxic mechanisms. Experimental studies have shown that aluminum exposure induces oxidative stress in neural tissues, promotes tau protein phosphorylation (a hallmark of neurodegenerative pathology), and disrupts glutamatergic and cholinergic neurotransmitter systems [13, 18]. Occupational studies of aluminum workers have demonstrated elevated oxidative stress markers that correlate with neurobehavioral impairments, including memory deficits and mood disturbances [18]. The combination of BaP and aluminum exposure may produce additive or synergistic neurotoxic effects, as both compounds activate overlapping inflammatory and oxidative stress pathways.

Anxiety is one of the most prevalent mental health disorders among industrial workers, characterized by persistent worry, fear, and physiological symptoms that can significantly impair work performance and diminish overall quality of life. While direct epidemiological studies linking BaP to anxiety in humans are still developing, the established biological pathway is highly relevant: systemic inflammation is a recognized contributor to the development of depressive and anxiety symptoms. Therefore, exposure to environmental toxicants like BaP and aluminum, which are known to increase systemic inflammation, could plausibly exacerbate anxiety symptoms by promoting neuroinflammation and disrupting neurotransmitter systems [13–15, 17].

Given the widespread nature of PAHs and aluminum co-exposure and the increasing prevalence of anxiety in industrial settings, understanding the psychological consequences of this exposure is crucial for improving worker health and safety. While previous experimental studies have demonstrated anxiety-like behaviors following BaP exposure in animal models [17] and occupational studies have extensively investigated PAH and aluminum exposure in aluminum smelters [6, 7, 13], few studies have simultaneously examined the association of both PAH and aluminum co-exposure with mental health outcomes in an occupational cohort. Furthermore, the relative contributions of chemical exposures versus psychosocial job stress to anxiety symptoms have not been adequately explored in this population. Therefore, this study aimed to investigate the associations of urinary 1-OHP and urinary aluminum levels with anxiety symptoms in aluminum workers, while controlling for job stress as a potential confounder. It is hypothesized whether the elevated levels of both biomarkers will correlate with higher anxiety scores or not. The study also compares anxiety levels between workers exposed to PAHs and aluminum and a control group to further explore the mental health impact of this exposure.

The results of this study could provide important insights into the psychological consequences of combined PAHs and aluminum exposure and help inform future strategies to mitigate occupational risks associated with these hazardous compounds. Furthermore, these findings may advocate for the integration of mental health screening into routine occupational health surveillance for workers exposed to these neurotoxicants.

## Methods

### Study design and participants

This cross-sectional study was conducted at an aluminum production facility. Sample size was calculated using G*Power software (version 3.1.9.7) for multiple linear regression with six independent variables, assuming a medium effect size (f² = 0.15), alpha = 0.05, and power = 0.80. This calculation yielded a minimum sample size of 98 participants. A total of 270 workers were invited to participate, of whom 260 (130 workers with occupational exposure to PAHs and aluminum and 130 unexposed controls) completed the study questionnaires and provided urine samples, yielding a response rate of 96.3%.

The exposed group was recruited from departments with historically high levels of PAH and aluminum dust exposure, including anode production, pot-rooms, and casting operations [6, 7]. The control group consisted of administrative and security personnel with no direct involvement in the primary smelting processes, ensuring negligible exposure to industrial PAHs and aluminum particulates. Controls were group-matched to the exposed workers based on age, gender (all male), and duration of employment to minimize confounding from these variables.

Smoking status was assessed through self-report during the health screening interview. Participants were classified as current smokers if they reported smoking at least one cigarette per day within the past month. Due to the relatively small number of smokers in each group (54 and 53 in exposed and control groups, respectively), smoking was included as a dichotomous variable (smoker/non-smoker) in the regression analysis.

### Inclusion and exclusion criteria

All participants were required to be full-time employees with a minimum of one year of continuous employment at the plant to ensure adequate duration for potential exposure and effect manifestation. Participants were excluded if they reported a pre-existing diagnosis of a major neurological disorder (e.g., epilepsy, neurodegenerative disease) or a major psychiatric condition (e.g., schizophrenia, bipolar disorder) as documented in their pre-employment or annual health screenings. This exclusion criterion was implemented to minimize the potential for confounding the association between exposure and anxiety symptoms. Furthermore, individuals on regular medication for anxiety or depression were also excluded to reduce the influence of pharmacological treatment on the outcome assessment. Additionally, we assessed self-reported chronic disease status (diabetes, cardiovascular disease, and respiratory conditions) during the health screening interview, and individuals with major chronic diseases were excluded to minimize confounding from these conditions.

### Chemicals

1-Hydroxypyrene was obtained from Sigma-Aldrich (St. Louis, MO, USA). Benzo[a]pyrene (BaP) was purchased from Dr. Ehrenstorfer GmbH (Augsburg, Germany). β-glucuronidase-arylsulfatase enzyme solution was supplied by Roche (Basel, Switzerland). Acetonitrile, benzene, cyclohexane, methylene chloride, Nitric acid (trace metal grade), aluminum standard solutions, and toluene (HPLC grade) were purchased from Merck (Darmstadt, Germany). C18 solid-phase extraction cartridges (1 mL) were obtained from Macherey-Nagel (Düren, Germany). PTFE membrane filters and pre-cleaned XAD-2 sorbent tubes were supplied by SKC (Eighty Four, PA, USA).

### BaP exposure assessment

Personal airborne exposure to benzo[a]pyrene (BaP) was assessed in accordance with NIOSH Method 5515 [19]. Air samples were collected using a sampling train consisting of a 2-µm PTFE filter followed by a washed XAD-2 sorbent tube to capture both particulate and vapor phases. Personal sampling pumps were calibrated to operate at 2 L/min for full-shift monitoring.

Samples were protected from light and shipped under cold conditions at approximately 4 °C using insulated cold boxes with ice packs. Analysis was performed using gas chromatography with flame ionization detection (GC-FID) following solvent extraction of the filter and sorbent media according to NIOSH Method 5515. Quality assurance procedures included analysis of field blanks and calibration with certified standards.

### Urinary aluminum analysis

Urine samples were collected at the end of the work shift on the last day of the work week to capture cumulative exposure over the work week. Samples were collected in sterile polypropylene containers, immediately placed on ice, and transported to the laboratory within 2 h of collection. Samples were stored at -20 °C until analysis. Urinary aluminum concentration was measured using inductively coupled plasma mass spectrometry (ICP-MS) following a validated method for multi-element biomonitoring in occupational settings [12]. Briefly, 1 mL of urine sample was diluted 1:10 with 0.5% nitric acid containing internal standards (yttrium and rhodium). Samples were analyzed using an Agilent 7900 ICP-MS (Agilent Technologies, Tokyo, Japan) operating in helium collision mode to eliminate polyatomic interferences. The instrument was calibrated using certified aluminum standards (0.1–100 µg/L). Analytical quality control was ensured through the analysis of certified reference urine control materials (ClinChek^®^ Urine Controls, RECIPE, Germany) and procedural blanks. The limit of detection for aluminum was 0.5 µg/L, and all samples were above this threshold.

### 1-Hydroxypyrene analysis

Urinary 1-hydroxypyrene (1-OHP) was analyzed following the procedure outlined previously [20]. In brief, 10 mL of urine was brought to a pH of 5.0 using 1 mol/L hydrochloric acid, after which 0.1 mol/L acetate buffer (pH 5.0) was added to reach a final volume of 30 mL. The mixture was incubated at 37 °C for 16 h in a shaking water bath after the addition of 15 mL of a glucuronidase–arylsulfatase enzyme solution.

For extraction, C18 reversed-phase cartridges were conditioned sequentially with 5 mL of methanol and 10 mL of distilled water. The enzymatically treated urine samples were loaded onto the cartridges at a flow rate of approximately 10 mL/min. The columns were rinsed first with 3 mL of distilled water and then with 3 mL of 50% methanol. Subsequently, 1-OHP was eluted using 8 mL of methanol. The eluate was evaporated to dryness and the residue was dissolved in 1 mL of methanol.

Quantification of 1-OHP was carried out using an Agilent 1200 series high-performance liquid chromatography system (Agilent Technologies, Folsom, CA, USA), equipped with a C18 reversed-phase column (150 × 4.6 mm; Agilent Technologies, CA, USA) and a fluorescence detector (Agilent Technologies, Waldbronn, Germany). The instrument was set to an excitation wavelength of 242 nm and an emission wavelength of 388 nm. Final urinary 1-OHP values were adjusted for creatinine concentration and reported as µmol/mol creatinine.

### Anxiety measurement

Anxiety was assessed using the Beck Anxiety Inventory (BAI), a widely used 21-item self-report questionnaire designed to measure the severity of anxiety symptoms [21]. Participants rated how much they were bothered by each common symptom of anxiety over the past week on a 4-point scale ranging from 0 (“not at all”) to 3 (“severely—I could barely stand it”). The total score was calculated by summing the scores of all 21 items. The BAI was developed to discriminate anxiety from depression and has demonstrated high internal consistency (Cronbach’s α = 0.92) and good test-retest reliability in various populations. The Persian version of the BAI has demonstrated strong psychometric properties, including acceptable test-retest reliability and internal consistency, supporting its use in this study [22].

### Job stress assessment

Job stress was evaluated using the Job Content Questionnaire (JCQ), a validated instrument grounded in the Demand-Control-Support model designed to measure key psychosocial job characteristics [23]. The JCQ core scales assess psychological job demands, decision latitude (job control), and workplace social support from both supervisors and coworkers. Respondents indicate their level of agreement with statements on a 4-point Likert scale (from “strongly disagree” to “strongly agree”). Prior to statistical analysis, the scores for the protective dimensions (job control and social support) were reversed, after which the total job stress score was derived by summing the scores of all dimensions.

The Persian (Farsi) version of the JCQ has been validated and shown to have strong psychometric properties among Iranian hospital nurses, confirming its reliability and validity for use in this occupational context [24].

### Statistical analysis

Descriptive statistics were used to summarize demographic characteristics, 1-OHP and aluminum concentrations, and BAI and job stress scores. Differences between exposed and control groups were analyzed using independent t-tests for continuous variables and chi-square tests for categorical variables. Pearson correlation coefficients were calculated to examine bivariate associations between exposure biomarkers, job stress, and anxiety. Multiple linear regression was employed to model the relationship between anxiety (dependent variable) and exposure biomarkers (1-OHP and urinary aluminum), while including age, job experience, smoking, and total job stress as independent variables.

All regression assumptions were assessed. Multicollinearity was assessed using the Variance Inflation Factor (VIF). The Durbin-Watson statistic was used to test for autocorrelation. Normality of residuals was assessed using the Shapiro-Wilk test and visual inspection of histograms and Q-Q plots. Influential points were assessed using Cook’s distance and leverage values. To further explore the relationship between chemical exposures, job stress, and anxiety, we conducted mediation analysis using the Baron and Kenny approach to assess whether job stress mediated the association between exposure biomarkers and anxiety. Cohen’s f² was calculated to assess effect size. Statistical significance was set at *p* < 0.05. All statistical analyses were performed using SPSS version 22.

#### Regression diagnostics

Multicollinearity was assessed using VIF (all values < 5 for primary variables). The Durbin-Watson statistic (2.011) indicated no autocorrelation. Visual inspection of residual plots supported linearity and homoscedasticity, and the Shapiro-Wilk test (*p* < 0.001) indicated some deviation from normality, which is acceptable given the large sample size (*N* = 260). Cook’s distance and leverage values indicated no influential outliers. Overall, these diagnostics suggest that the regression model meets the assumptions for valid inference.

#### Mediation analysis

To further examine the relationship between chemical exposures, job stress, and anxiety, we performed mediation analysis using the Baron & Kenny approach. Job stress did not mediate the association between 1-OHP and anxiety (β = 0.039, *p* = 0.683).

## Results

### Participant characteristics and group comparisons

**Fig. 1:**
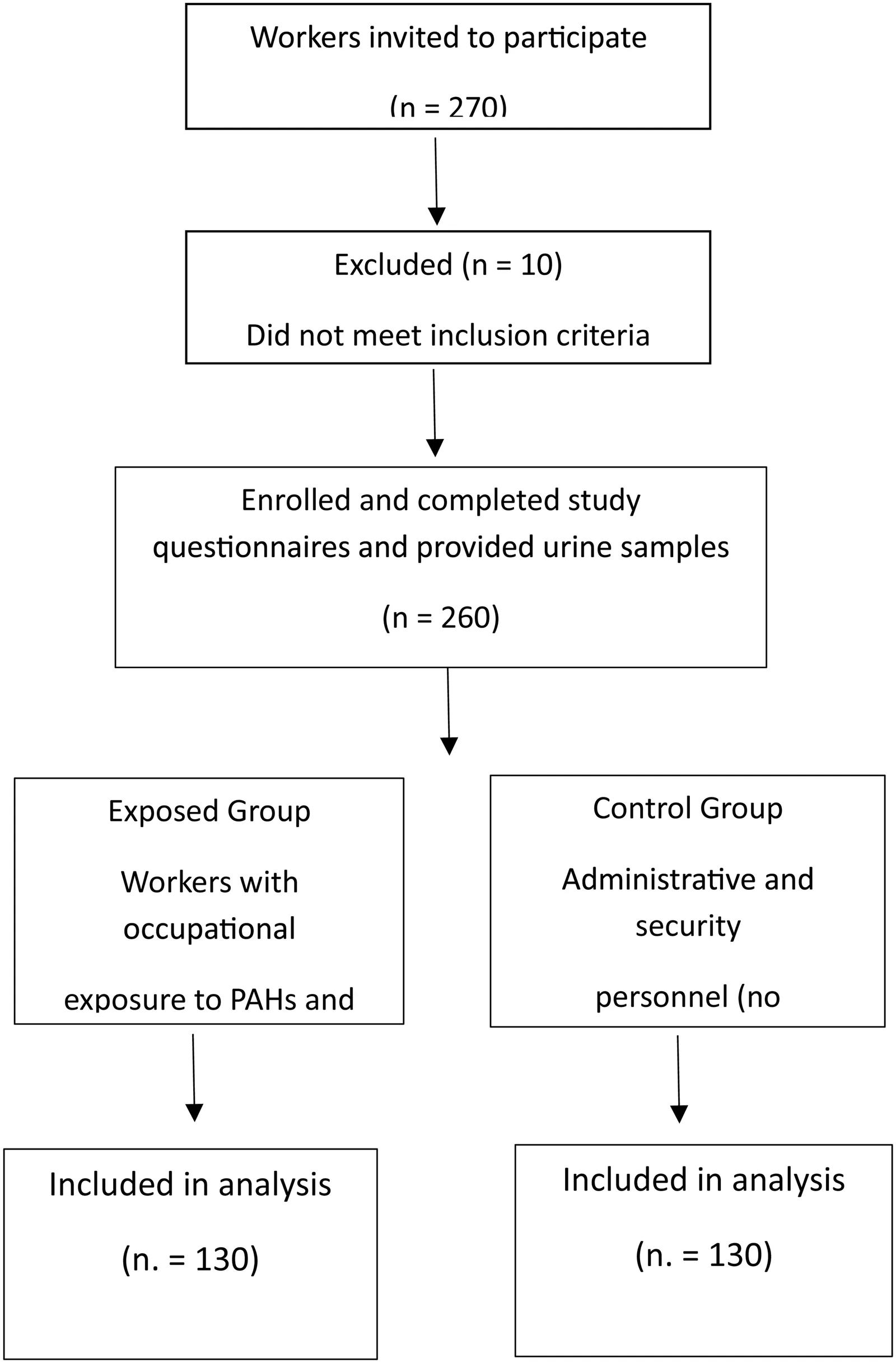
Participant flow diagram showing recruitment, enrollment, and analysis of study participants

Figure 1 presents the participant flow diagram, showing the recruitment and enrollment of 260 participants (130 exposed workers and 130 matched controls) included in the final analysis. Among the 260 participants, 54 and 53 smokers were in the exposed and control groups, respectively. There was no significant difference in smoking prevalence between groups (χ² = 0.016, *p* = 0.900).

Independent samples t-tests revealed no statistically significant differences between the exposed and control groups in terms of age (*p* = 0.236) or job experience (*p* = 0.955). However, the exposed group had significantly higher total job stress scores (52.45 ± 12.72) compared to the control group (47.45 ± 11.25), with a mean difference of 5.01 (95% CI [2.07, 7.94], t (258) = 3.362, *p* = 0.001) (Table 1).

The exposed group had significantly higher levels of airborne BaP (2.27 ± 1.68 µg/m³) compared to the control group (< LOD; treated as 0.00 µg/m³ for statistical analysis), with a mean difference of 2.27 µg/m³ (95% CI [1.98, 2.56], t(258) = 15.400, *p* < 0.001). Similarly, urinary 1-hydroxypyrene was significantly higher in the exposed group (7.30 ± 4.69 µmol/mol creatinine) than in the control group (2.59 ± 2.89 µmol/mol creatinine), representing a mean difference of 4.70 µmol/mol creatinine (95% CI [3.75, 5.65], t(258) = 9.743, *p* < 0.001) (Table 1).

Critically, urinary aluminum concentration was significantly higher in the exposed group (33.45 ± 16.20 µg/g creatinine) compared to controls (7.35 ± 3.99 µg/g creatinine), representing a mean difference of 26.10 µg/g creatinine (95% CI [23.22, 28.98], t (258) = 17.837, *p* < 0.001).

The mean anxiety score was significantly higher in the exposed group (10.74 ± 6.18) compared to the control group (8.88 ± 5.28), a difference that was statistically significant (mean difference = 1.86, 95% CI [0.46, 3.27], t (258) = 2.611, *p* = 0.010).

**Table 1:** Characteristics and comparison of exposed and control groups for workers in an aluminum production facility, Iran (March 2025)

Variable	Exposed Group (*n* = 130) Mean ± SD	Control Group (*n* = 130) Mean ± SD	Mean Difference [95% CI]	*p*-value
Age (years)	42.47 ± 7.46	41.43 ± 6.63	1.04 [-0.68, 2.76]	0.236
Job Experience (years)	17.62 ± 7.23	17.58 ± 6.02	0.05 [-1.58, 1.67]	0.955
BaP (µg/m³)	2.27 ± 1.68	< LOD	2.27 [1.98, 2.56]	< 0.001*
1-Hydroxy Pyrene (µmol/mol creatinine)	7.30 ± 4.69	2.59 ± 2.89	4.70 [3.75, 5.65]	< 0.001*
Urinary Aluminum (µg/g creatinine)	33.45 ± 16.20	7.35 ± 3.99	26.10 [23.22, 28.98]	< 0.001*
Anxiety Score	10.74 ± 6.18	8.88 ± 5.28	1.86 [0.46, 3.27]	0.010*
Total Job Stress Score	52.45 ± 12.72	47.45 ± 11.25	5.01 [2.07, 7.94]	0.001*

*Statistically significant (*p* < 0.05)

### Correlation analysis

Pearson correlation analysis revealed that anxiety was significantly correlated with all exposure and psychosocial variables. Urinary 1-hydroxypyrene showed a strong positive correlation with anxiety (*r* = 0.505, *p* < 0.001). Urinary aluminum demonstrated a moderate positive correlation with anxiety (*r* = 0.417, *p* < 0.001). Total job stress score was significantly correlated with anxiety (*r* = 0.435, *p* < 0.001). Airborne BaP showed a weaker but significant correlation with anxiety (*r* = 0.249, *p* < 0.001). Notably, urinary 1-hydroxypyrene and urinary aluminum were moderately correlated with each other (*r* = 0.564, *p* < 0.001), and both were significantly correlated with total job stress (*r* = 0.259 and *r* = 0.370, respectively, *p* < 0.001) (Table 2).

**Table 2:** Pearson correlation coefficients between exposure biomarkers, job stress, and anxiety

Variable	1	2	3	4	5
1. BaP (µg/m³)	1				
2. 1-Hydroxypyrene (µmol/mol creatinine)	0.629**	1			
3. Anxiety	0.249**	0.505**	1		
4. Urinary Aluminum (µg/g creatinine)	0.596**	0.564**	0.417**	1	
5. Total Job Stress Score	0.235**	0.259**	0.435**	0.370**	1

**Correlation is significant at the 0.01 level (2-tailed). *N* = 260

### Multiple linear regression analysis

Given that urinary 1-hydroxypyrene showed a stronger bivariate correlation with anxiety (*r* = 0.505, *p* < 0.001) compared to airborne BaP (*r* = 0.249, *p* < 0.001), and due to the high correlation between BaP and 1-OHP (*r* = 0.629, *p* < 0.001) which would introduce multicollinearity, airborne BaP was not included in the final regression model. Therefore, 1-OHP was retained as the preferred PAHs exposure metric. The regression analysis included six independent variables: urinary 1-hydroxypyrene, urinary aluminum, smoking, total job stress score, age, and job experience. The model was statistically significant, explaining 38.4% of the variance in anxiety scores (R² = 0.384, adjusted R² = 0.369, F (6, 253) = 26.290, *p* < 0.001). The effect size was large (Cohen’s f² = 0.623). In the final regression model, which included total job stress as an independent variable alongside age, job experience, and smoking, urinary 1-OHP (β = 0.204, *p* = 0.013), urinary aluminum (β = 0.177, *p* = 0.009), smoking (β = 0.202, *p* = 0.003), and total job stress (β = 0.295, *p* < 0.001) were all significantly associated with anxiety, while Age (*p* = 0.245) and job experience (*p* = 0.298) did not show statistically significant associations with anxiety (Table 3).

**Table 3:** Multiple linear regression results for variables associated with anxiety scores

Independent variables	Unstandardized B	Std. Error	Standardized β	*p*-value	95% CI for B
(Constant)	2.355	2.916		0.420	[-3.391, 8.101]
Age	-0.119	0.102	-0.144	0.245	[-0.320, 0.082]
Job experience	0.113	0.108	0.129	0.298	[-0.101, 0.327]
Total Job Stress Score	0.140	0.025	0.295	< 0.00*1	[0.090, 0.190]
1-Hydroxypyrene (µmol/mol creatinine)	0.260	0.105	0.204	0.013*	[0.054, 0.466]
Smoking	2.386	0.804	0.202	0.003*	[0.801, 3.971]
Urinary Aluminum (µg/g creatinine)	0.058	0.022	0.177	0.009*	[0.014, 0.102]

Dependent Variable: Anxiety Score. R² = 0.384, Adjusted R² = 0.369, F(6, 253) = 26.290, *p* < 0.001, Cohen’s f² = 0.623

*Statistically significant (*p* < 0.05)

For urinary aluminum, although the path to job stress was significant (β = 0.351, *p* < 0.001), the direct effect of aluminum on anxiety remained significant when job stress was included in the model (Table 3: β = 0.177, *p* = 0.009), indicating no mediation. These findings indicate that both chemical exposures are independently associated with anxiety, and job stress acts as an additional independent factor rather than as a mediator.

## Discussion

This cross-sectional study investigated the associations of occupational exposure to PAHs (measured via urinary 1-OHP) and aluminum with anxiety symptoms among workers in an aluminum production plant. The principal finding of our research is that both urinary 1-OHP and urinary aluminum levels demonstrated significant, positive associations with anxiety scores, in a model that included total job stress as an independent variable alongside age, job experience, and smoking. This association was robust and explained a substantial portion of the variance in anxiety (R² = 0.384).

Our group comparisons confirmed the fundamental premise of the study, showing that workers in high-exposure areas had significantly elevated levels of BaP, 1-OHP, and urinary aluminum. Crucially, these exposed workers also reported significantly higher anxiety scores than their non-exposed counterparts. Notably, the exposed group also reported significantly higher total job stress scores compared to the control group (*p* = 0.001), suggesting that psychosocial factors beyond chemical exposure may also contribute to psychological distress in this population.

The core finding that urinary 1-OHP and urinary aluminum are significantly associated with anxiety provides a critical advancement beyond simply documenting exposure. While 1-OHP is a well-validated biomarker for total PAH exposure [10] and urinary aluminum is a validated biomarker of aluminum body burden [12], their specific correlation with a mental health outcome in an occupational cohort is a significant contribution. This result implies that the internal doses of PAHs and aluminum, as reflected by these biomarkers, are biologically relevant to neurological and emotional function. This aligns with a growing body of evidence from both animal and human studies indicating that PAHs like BaP are potent neurotoxicants, primarily through proposed mechanisms such as the induction of oxidative stress and neuroinflammation [14, 15]. Similarly, aluminum induces oxidative stress, promotes tau protein phosphorylation, and disrupts glutamatergic neurotransmission, all of which may contribute to anxiety-like behaviors [13, 18]. The co-exposure to both agents may produce additive or synergistic neurotoxic effects through shared pathways of neuroinflammation and blood-brain barrier disruption.

Human epidemiological studies investigating the relationship between PAH exposure and mental health outcomes are still limited; however, emerging evidence supports a potential association. For instance, the GuLF STUDY cohort [25] demonstrated that workers engaged in oil spill clean-up operations, who were exposed to PAHs through oil vapors and chemical dispersants, exhibited significantly elevated depression and anxiety scores relative to the general population. Although such studies are constrained by their cross-sectional design and the potential for residual confounding, their findings are consistent with our observation of a significant association between PAH exposure and anxiety symptoms among aluminum workers. Our study contributes to this growing body of literature by utilizing validated biomarkers of PAH exposure (urinary 1-OHP) and explicitly controlling for job stress as a potential psychosocial confounder, thereby providing more robust evidence for this association.

An important finding of this study is that total job stress had the strongest statistical association with anxiety in the multivariate model (β = 0.295, *p* < 0.001), even when included alongside chemical exposures. This finding is consistent with a large body of occupational health literature demonstrating that psychosocial work factors, particularly job strain, are significant contributors to mental health outcomes [26, 27]. The fact that both chemical exposures (PAHs and aluminum) and psychosocial stress showed significant associations with anxiety suggests that these factors may have additive effects on mental health. Our mediation analysis revealed that job stress does not mediate the association between chemical exposures and anxiety. Rather, both chemical exposures (PAHs and aluminum) and job stress have independent and additive associations with anxiety. This suggests that the neurotoxic effects of PAHs and aluminum on anxiety are direct and not explained by psychosocial pathways. The correlation analysis further supports this interpretation. As shown in Table 2, both chemical exposure biomarkers and total job stress were significantly correlated with anxiety, with 1-OHP showing the strongest bivariate correlation (*r* = 0.505) followed by job stress (*r* = 0.435) and urinary aluminum (*r* = 0.417). The significant correlations between job stress and exposure biomarkers (*r* = 0.259 with 1-OHP, *r* = 0.370 with urinary aluminum) suggest that chemical exposure and psychosocial stress co-occur in this occupational setting, potentially through shared occupational factors such as department assignment, shift work, and physical work demands. This co-occurrence may reflect the clustering of multiple risk factors in more demanding and hazardous work environments. The significant association of both chemical exposures and job stress in the multivariate model, however, support an additive effect on anxiety symptoms.

Regarding the choice of PAH exposure metric, airborne BaP was not included in the final regression model. As shown in the correlation matrix, urinary 1-OHP demonstrated a stronger correlation with anxiety (*r* = 0.505) compared to airborne BaP (*r* = 0.249). Additionally, the high correlation between BaP and 1-OHP (*r* = 0.629) would introduce multicollinearity if both were included. These findings support the use of urinary 1-OHP as the preferred biomarker for assessing PAH-related neuropsychological effects, as it reflects the internal dose of total PAHs rather than external exposure to a single compound [10].

The finding that smoking was significantly associated with anxiety (β = 0.202, *p* = 0.003) is consistent with previous research demonstrating that tobacco smoke is a major source of PAHs and other neurotoxicants, and that smoking behavior is associated with anxiety symptoms [28, 29]. This association remained significant even when occupational PAH exposure (1-OHP) was included in the model, suggesting that smoking contributes additional neurotoxic burden beyond occupational sources.

### Clinical significance

The mean difference in anxiety scores between exposed and control groups (1.86 points on the BAI) is statistically significant (p = 0.010) but modest in absolute terms. Both groups’ mean scores fall within the ‘mild’ anxiety range (8–15 on the BAI), with the exposed group at 10.74 and the control group at 8.88. While this difference may not represent clinically significant anxiety in the majority of individuals, several considerations are important. First, the distribution of anxiety scores was wider in the exposed group (SD = 6.18) compared to controls (SD = 5.28), with some exposed workers scoring in the moderate-to-severe range (BAI > 26). These individuals represent a subgroup of particular concern who may benefit from targeted screening and intervention. Second, the significant association of 1-OHP and urinary aluminum in the regression model (β = 0.204 and 0.177, respectively) indicate that these exposures contribute to anxiety symptoms across the population, even if the group-level difference is modest. Third, occupational exposures accumulate over many years. While the cross-sectional difference between groups is modest at a single time point, cumulative exposure over decades may lead to progressively larger differences in mental health outcomes. Fourth, even subclinical anxiety can affect work performance, quality of life, and overall well-being. In occupational settings, early identification of individuals at risk may allow for preventive interventions before symptoms reach clinical significance. Thus, while the clinical significance of the group-level difference should not be overstated, our findings support the use of biological monitoring as part of a comprehensive approach to occupational mental health surveillance.

### Limitations

Several limitations should be acknowledged. The cross-sectional design prevents causal inference, and exposure biomarkers were assessed at a single time point, which may not fully capture long-term or cumulative exposure patterns. Although we controlled for key confounders, residual confounding from unmeasured factors such as education, socioeconomic status, shift work, sleep quality, alcohol consumption, BMI, and chronic diseases cannot be ruled out, and we recommend that future studies incorporate these variables to further isolate the independent effects of chemical exposures. The use of self-reported anxiety measures may be subject to reporting biases. Additionally, we cannot rule out the healthy worker effect, as workers who developed severe health effects may have left employment or been transferred to lower-exposure departments. The study was conducted at a single aluminum plant and included only male workers, limiting generalizability to other settings, industries, or female workers.

## Conclusion

Our study provides evidence of a significant association between biomarkers of PAH exposure and aluminum exposure and anxiety in a well-defined occupational population. Notably, job stress was also strongly associated with anxiety, highlighting the multifactorial nature of psychological distress in industrial settings. The fact that both chemical and psychosocial factors independently contributed to anxiety suggests that comprehensive occupational health interventions should address both domains.

These results have important implications for occupational health practices. They suggest that biomonitoring of 1-OHP and urinary aluminum could serve a dual purpose: not only for assessing cancer risk and metal body burden but also for identifying populations at potential risk for psychological distress. Furthermore, given the strong association between job stress and anxiety, interventions aimed at reducing psychosocial work stressors should be implemented alongside efforts to reduce chemical exposures. This warrants the consideration of integrating mental health screening and support services into the health surveillance protocols of industries with high PAHs and aluminum co-exposure. However, future research employing longitudinal designs is needed to confirm causality and further elucidate the specific neurobiological mechanisms linking combined PAH and aluminum exposure to anxiety and other mental health disorders.

## Supplementary Information

Below is the link to the electronic supplementary material.

Supplementary Material 1

Supplementary Material 2

## Data Availability

All data supporting the findings of this study are available within the paper.

## Abbreviations

1-OHP: 1-hydroxypyrene
BaP: Benzo[a]pyrene
BAI: Beck Anxiety Inventory
GC-FID: Gas Chromatography with Flame Ionization Detection
HPLC: High-Performance Liquid Chromatography
ICP-MS: Inductively Coupled Plasma Mass Spectrometry
JCQ: Job Content Questionnaire
LOD: Limit of Detection
PAHs: Polycyclic Aromatic Hydrocarbons
VIF: Variance Inflation Factor
