# Supplementary material for: Assessment of anxiety risk association with PAHs and aluminum co-exposure in an aluminum production facility
Source: J Egypt Public Health Assoc. 2026 Sep 17;101:27. doi: 10.1186/s42506-026-00233-2 (PMC13586090; doi:10.1186/s42506-026-00233-2)
Supplement: Supplementary file 1 [file 42506_2026_233_MOESM1_ESM.pdf]

Great Plains Health
Behavioral Health

Beck Anxiety Inventory (BAI)

About: This scale is a self-report measure of anxiety.

Items: 21

Reliability:
Internal consistency for the BAI = (Cronbach's a=0.92)
Test-retest reliability (1 week) for the BAI = 0.75 (Beck, Epstein, Brown, & Steer, 1988)

Validity:
The BAI was moderately correlated with the revised Hamilton Anxiety Rating Scale (.51), and mildly correlated with the Hamilton Depression Rating Scale (.25) (Beck et al., 1988).

Scoring:

	Not at all	Mildly, but it didn't bother me much	Moderately – it wasn't pleasant at times	Severely – it bothered me a lot
All questions	0	1	2	3

The total score is calculated by finding the sum of the 21 items.
Score of 0-21 = low anxiety
Score of 22-35 = moderate anxiety
Score of 36 and above = potentially concerning levels of anxiety

References: Beck, A.T., Epstein, N., Brown, G., & Steer, R.A. (1988). An inventory for measuring clinical anxiety: Psychometric properties. *Journal of Consulting and Clinical Psychology*, 56, 893-897.

Beck Anxiety Inventory (BAI)

Below is a list of common symptoms of anxiety. Please carefully read each item in the list. Indicate how much you have been bothered by that symptom during the past month, including today, by circling the number in the corresponding space in the column next to each symptom.

	Not at all	Mildly, but it didn't bother me much	Moderately – it wasn't pleasant at times	Severely – it bothered me a lot
Numbness or tingling	0	1	2	3
Feeling hot	0	1	2	3
Wobbliness in legs	0	1	2	3
Unable to relax	0	1	2	3
Fear of worst happening	0	1	2	3
Dizzy or lightheaded	0	1	2	3
Heart pounding / racing	0	1	2	3
Unsteady	0	1	2	3
Terrified or afraid	0	1	2	3
Nervous	0	1	2	3
Feeling of choking	0	1	2	3
Hands trembling	0	1	2	3
Shaky / unsteady	0	1	2	3
Fear of losing control	0	1	2	3
Difficulty in breathing	0	1	2	3
Fear of dying	0	1	2	3
Scared	0	1	2	3
Indigestion	0	1	2	3
Faint / lightheaded	0	1	2	3
Face flushed	0	1	2	3
Hot / cold sweats	0	1	2	3
