# Supplementary material for: Assessment of anxiety risk association with PAHs and aluminum co-exposure in an aluminum production facility
Source: J Egypt Public Health Assoc. 2026 Sep 17;101:27. doi: 10.1186/s42506-026-00233-2 (PMC13586090; doi:10.1186/s42506-026-00233-2)
Supplement: Supplementary file 2 [file 42506_2026_233_MOESM2_ESM.pdf]

Job Content Questionnaire (JCQ) Summary with Scores

Dimension	Subscale	Description	Example Questions	Number of Items	Min Score	Max Score
**Job Demands**	Psychological Demands	Measures mental workload and time pressure at work.	- My job requires working very fast.<br>- My job requires me to work very hard.<br>- I have enough time to get the job done (*reverse coded*).<br>- I am asked to do an excessive amount of work.	5	5	20
**Job Control**	Decision Latitude	Evaluates the degree of control employees have over their work, including decision-making and skill use.	- I have the freedom to decide how to do my job.<br>- I have a lot of say in decisions about my work.<br>- I have control over what happens in my job.	4	4	16
	Skill Discretion	Assesses opportunities for creativity, skill utilization, and professional growth.	- My job requires a high level of skill.<br>- I have the opportunity to develop my abilities.<br>- My job involves a lot of repetitive tasks (*reverse coded*).	5	5	20

Dimension	Subscale	Description	Example Questions	Number of Items	Min Score	Max Score
**Social Support**	Supervisor Support	Assesses the level of support and encouragement from supervisors.	- My supervisor is helpful in getting the job done.<br>- My supervisor pays attention to what I say.<br>- I can talk to my supervisor about problems at work.	4	4	16
	Coworker Support	Evaluates the level of collaboration and assistance among colleagues.	- People I work with are competent in doing their jobs.<br>- I get along well with my coworkers.<br>- My coworkers are helpful in completing tasks.	4	4	16
**Physical Environment**	Physical Job Demands	Evaluates physical aspects of the job, such as lifting, repetitive tasks, or exposure to hazards.	- My job requires lots of physical effort.<br>- My job involves exposure to hazardous conditions.<br>- My job requires frequent lifting or carrying of heavy loads.	3	3	12

Notes on Scoring:

1. **Psychological Demands**: Higher scores indicate higher mental workload and time pressure (i.e., higher stress).
2. **Job Control**: Higher scores indicate greater autonomy and decision-making ability (i.e., lower stress).
3. **Social Support**: Higher scores indicate stronger support from coworkers and supervisors (i.e., lower stress).
4. **Physical Job Demands**: Higher scores indicate more physically demanding work conditions (i.e., higher stress).

For each dimension, the **minimum score** is calculated by assigning a score of **1** to all items, and the **maximum score** is calculated by assigning a score of **4** to all items.

To meet your request fully, here's a detailed table for the **Job Content Questionnaire (JCQ)** with all questions for each dimension, considering the number of items specified for each subscale:

Dimension	Subscale	Description	Example Questions	Number of Items	Min Score	Max Score
**Job Demands**	**Psychological Demands**	Measures mental workload and time pressure at work.	- My job requires working very fast.<br>- My job requires working very hard.<br>- My job is very hectic.<br>- I am free from conflicting demands that others make.<br>- I have enough time to get the job done (reverse coded).	5	5	20
**Job Control**	**Decision Latitude**	Evaluates the degree of control employees have over their work, including decision-making and skill use.	- My job allows me to make a lot of decisions on my own.<br>- I have a lot to say about what happens on my job.<br>- My job allows me to take part in making decisions that affect me.<br>- My job allows me to work independently.	4	4	16
	**Skill Discretion**	Assesses opportunities for creativity, skill utilization, and professional growth.	- My job requires a high level of skill.<br>- My job requires a variety of different things to be done.<br>- My job requires	5	5	20

Dimension	Subscale	Description	Example Questions	Number of Items	Min Score	Max Score
			me to learn new things.<br>- I have the opportunity to develop my abilities.<br>- My job involves a lot of repetitive tasks (reverse coded).			
**Social Support**	**Supervisor Support**	Assesses the level of support and encouragement from supervisors.	- My supervisor is helpful in getting the job done.<br>- My supervisor pays attention to what I say.<br>- My supervisor is concerned about the welfare of those under them.<br>- I can talk to my supervisor about problems at work.	4	4	16
	**Coworker Support**	Evaluates the level of collaboration and assistance among colleagues.	- People I work with are competent in doing their jobs.<br>- People I work with are friendly.<br>- I get along well with my coworkers.<br>- My coworkers are helpful in completing tasks.	4	4	16
**Physical Environment**	**Physical Job Demands**	Evaluates physical aspects of the job, such as lifting, repetitive tasks, or exposure to hazards.	- My job requires lots of physical effort.<br>- My job involves exposure to hazardous	5	5	20

Dimension	Subscale	Description	Example Questions	Number of Items	Min Score	Max Score
			conditions.<br>- My job requires frequent lifting or carrying of heavy loads.<br>- My job requires working in uncomfortable positions.<br>- My job is physically demanding.			
